# Peripheral nerve stimulation for the treatment of anterior cutaneous nerve entrapment syndrome: A case report and literature review

**DOI:** 10.1016/j.inpm.2025.100653

**Published:** 2025-11-27

**Authors:** Royce Copeland, Yacoub Khatab, Ravinderjit Singh, Emanuel N. Husu

**Affiliations:** a Department of Pain Medicine, Division of Anesthesia, Critical Care and Pain Medicine, The University of Texas, MD Anderson Cancer Center, Houston, TX, USA; b Baylor College of Medicine, H. Ben Taub Department of Physical Medicine and Rehabilitation, Houston, TX, USA

**Keywords:** Anterior cutaneous nerve entrapment syndrome, Abdominal cutaneous nerve entrapment syndrome, Peripheral nerve stimulation, Neuromodulation

## Abstract

**Background:**

Anterior cutaneous nerve entrapment syndrome (ACNES) is a neuropathic pain condition characterized by irritation or compression of abdominal wall intercostal nerve branches called the anterior cutaneous nerves. Peripheral nerve stimulation (PNS) has become an effective treatment option for painful sensory neuropathic conditions, including mononeuropathies and nerve entrapment syndromes. This report describes a successful case of using temporary PNS to treat ACNES and reviews the available literature on the use of PNS for the treatment of ACNES.

**Case presentation:**

A 38-year-old female with a complex abdominal medical and surgical history presented to the pain medicine clinic for intractable burning pain and pressure in the left upper quadrant of the periumbilical region. Given the patient's clinical history and the nature of her presenting symptoms, ACNES was considered as a potential diagnosis. The diagnosis was confirmed through a series of successful diagnostic rectus sheath nerve blocks. A two-month temporary peripheral nerve stimulator trial targeting the left anterior cutaneous nerve was completed, and it resulted in 80 % pain reduction at 3 and 6-month follow-up evaluations, with returning pain at the 8-month assessment.

**Conclusion:**

Chronic abdominal pain in patients with a complex history of abdominal surgery should alert pain specialists to consider the possibility of an ACNES diagnosis. Current evidence supporting PNS for ACNES is limited to a small number of case reports showing successful treatment; however, larger-scale and more robust studies are needed to determine the effectiveness and safety of this method. This study contributes to the existing body of literature, highlighting that PNS may serve as a valuable treatment option for individuals with chronic abdominal wall pain secondary to ACNES whose pain is refractory to conservative management strategies.

## 1 Introduction

Anterior cutaneous nerve entrapment syndrome (ACNES), also known as abdominal cutaneous entrapment syndrome, represents a frequently underrecognized source of chronic abdominal wall pain and has been described as the “forgotten diagnosis.” [1]. ACNES arises from the compression of sensory nerves, specifically the anterior cutaneous branches of the 7th to 12th thoracic intercostal nerves [2]. These nerves pass between the internal oblique and transversus abdominis muscles (forming the posterior rectus sheath) before exiting this plane, proceeding to the posterior lateral aspect of the rectus abdominis muscle, where they travel through a fibrous neurovascular canal within this muscle before innervating the skin [2,3]. Ultimately, the cutaneous branches of the thoracoabdominal intercostal nerves are entrapped at the lateral border of the rectus abdominis muscle, resulting in severe, often refractory chronic pain [4].

Abdominal wall pain is responsible for up to 30 % of chronic abdominal pain cases, with ACNES being the most common underlying cause [10]. The prevalence of the syndrome ranges from 15 to 30 % depending on the definition and the diagnostic criteria used [2]. In 2015, a retrospective Dutch study found that approximately 2 % of patients presenting to the emergency department with acute abdominal pain were diagnosed with ACNES [3]. The onset of ACNES is often idiopathic, but a significant risk factor for this disorder is a history of abdominal trauma or surgical procedures, which result in distal nerve irritation or scarring at the entry points of the anterior cutaneous branches into the abdominal wall fascia [5]. Some authors have suggested that the entrapment may be caused by muscle contraction near the fibrous neurovascular canals, leading to mechanical or ischemic irritation resulting in severe abdominal wall pain [3,5,6].

The diagnosis of ACNES is based upon patient history and physical examination, thus making it challenging for physicians to identify the source of pain and provide the patient with effective treatment options. Past medical histories, symptoms, and exam findings often can lead to misidentification of various painful conditions, including diabetic radiculopathy, abdominal wall endometriosis, inflammatory bowel disease, abdominal wall tear, slipping rib syndrome, iliohypogastric or iliocostal impingement syndrome, abdominal wall hernia, or thoracic radiculopathy, which are potential causes of chronic abdominal wall pain [2,4,20]. However, the most common clinical scenario is an individual who reports localized, sharp, or burning abdominal wall pain along the rectus abdominis accompanied by altered sensation, such as hyperesthesia or hypoesthesia. The pain is usually situated lateral to the periumbilical region and is exacerbated by positional changes that stress abdominal muscles (coughing, body stretching, bowel movements, exercising) [7]. Key clinical features that aid in the diagnosis include a positive Carnett's sign and a positive response to a rectus sheath block(>50 % pain reduction) [7,8]. Carnett test is an important test to distinguish between parietal and visceral origins of abdominal pain. The clinician will identify the area of maximum tenderness on the abdominal wall localized by their finger, then have the patient lift their legs and head off the bed. If the pain is at least the same or worse, the test is considered positive and originates from the abdominal wall [8,11]. The rectus sheath block is a key component of the diagnosis of the ACNES as it provides midline somatic analgesia of the abdominal wall [9]. This diagnostic block should be performed between the rectus abdominis and the posterior rectus sheath. The T7-12 nerves travel through the transverse abdominis plane and enter deep to the rectus abdominis muscle, which ascends through the lateral half of the muscle to innervate tissues along the way to the ventral surface of the body [9]. There are two techniques: landmark and ultrasound-guided; however, the literature increasingly recommends ultrasound-guided local anesthetic injection. This method enables precise placement of the anesthetic and allows for visualization of potentially hazardous structures, such as the peritoneum or deep inferior epigastric artery that neighbor the target site [9]. Lastly, the diagnosis of ACNES is further supported when the patient has negative laboratory and imaging tests for intra-abdominal pathology.

Initial management of ACNES typically involves medical and interventional options. A variety of medications including muscle relaxers, nonsteroidal anti-inflammatories, tricyclic antidepressants, gabapentinoids, topic lidocaine or capsaicin creams; however, the reported effectiveness in providing adequate pain relief is frequently limited [11]. Interventional techniques include local anesthetic trigger point injections or rectus sheath blocks with or without corticosteroids, pulsed radiofrequency ablation, and botulinum toxin all with varying outcomes [1,10,11]. The most common interventional technique is the ultrasound or landmark guided local anesthetic trigger point injection technique [10.] A randomized, non-blinded multicenter clinic trial published in 2021 showed ultrasound versus landmark guidance had similar pain reduction outcomes at the 3-month evaluation following 10 mL 1 % lidocaine every two weeks for four injection sessions [23]. For patients with refractory ACNES, surgical neurectomy has historically been an effective treatment, offering long-term pain relief in approximately 70–90 % of appropriately selected cases [12]. Nevertheless, patients often prefer surgery as a last resort option and this surgical approach carries inherent risks such as infection, scarring, or sensory deficits.

Recently, PNS has emerged as a promising minimally invasive treatment for chronic neuropathic pain management, such as occipital neuralgia, cluneal neuralgia, poststroke shoulder pain, chronic pelvic pain, and several other neuropathies involving a single peripheral nerve [13,14]. While PNS has shown considerable promise, the available data specifically for its application in ACNES are currently limited, and its long-term effectiveness has not been evaluated. The aim of this literature review was to identify and critically evaluate PNS as a possible treatment option for ACNES and illustrate a case report with the successful application of PNS in managing this condition.

## 2 Materials and methods

The authors performed a comprehensive literature search in various databases (PubMed and Google Scholar) in order to identify the studies involving the treatment of ACNES with PNS. The following search terms were conducted in the databases alone or in various combinations: anterior cutaneous nerve entrapment syndrome, abdominal cutaneous nerve entrapment syndrome, ACNES, and peripheral nerve stimulation with filters limited to human studies from January 1st, 2000 to July 1st, 2025. The initial search yielded a total of 41 references from the combined databases. Duplicate articles were excluded, and abstracts were screened by two authors (YK & RC) pertaining to PNS for the treatment of ACNES. Articles not written in English or non-full manuscripts were excluded from the study. Two cases of peripheral nerve stimulation for the treatment of ANCES were identified and described in Table 1, which included 2 case reports. Written informed consent was obtained from the participant for publication of this case report and the accompanying images. The Institutional Review Board approval was waived.

**Table 1:** Study characteristics.

Study	Age	Sex	Type of Implant	Prior abdominal trauma or surgeries	Follow-up	Outcome
Ferreira-Silva et al., 2022	23	Female	StimRouter (permanent)	None	6 months	9/10 to 4/10 NRS 55 % pain reduction
Amorizzo et al., 2024	41	Male	StimRouter (permanent)	Not Reported	2 years	Not Reported “back to normal health”

## 3 Case Presentation

### 3.1 Patient history

A 38-year-old female with a complex abdominal surgical history, including cholecystectomy, gastrostomy tube (G-tube) placement, pyloroplasty, and gastrointestinal pacemaker implantation for severe gastroparesis, presented to the pain medicine clinic with a complaint of intolerable burning pain and pressure localized in the left upper quadrant abdominal region near the periumbilical region with sensory changes. The patient reported a history of percutaneous endoscopic gastrostomy (PEG) site infections and G-tube malfunctions, which were managed with oral antibiotics and tube replacements throughout the year before the initial presentation. The onset of the current pain, which is primarily localized above the insertion site, coincided with these subsequent G-tube replacements.

### 3.2 Prior treatments

Prior to the initial presentation at the pain medicine clinic, the patient's abdominal pain was being managed by her primary care physician and gastroenterologist. The patient had insufficient pain relief despite trialing a variety of medications, including rifaximin, gabapentin, duloxetine, dicycloverine, and methocarbamol.

### 3.3 Initial evaluation

Upon initial evaluation, the patient described severe left upper quadrant and epigastric abdominal pain, with the primary area of discomfort located above her G-tube site. The pain was exacerbated by activities involving flexion of her left hip or contraction of the abdomen. Additionally, valsalva maneuvers, such as straining during bowel movements, worsened her pain. She characterized the pain as burning and stabbing and as a deep pressure sensation, which significantly limited her ability to walk and play with her children. Physical examination demonstrated normal motor strength (5/5) in all extremities. Increased sensitivity to light touch was noted in the left upper quadrant and epigastric region, specifically above the G-tube insertion site. A positive Carnett's sign was elicited with pain predominantly localized in the same distribution. Deep tendon reflexes were normal, and upper motor neuron signs were absent.

### 3.4 Diagnostic work-up

To further investigate the cause of the patient's abdominal pain, a computed tomography (CT) scan of the abdomen and pelvis was performed. The study revealed prior cholecystectomy, without evidence of dilated bowel loops, free air, intra-abdominal inflammatory changes, or gastric wall thickening was noted. The percutaneous gastrostomy tube was visualized within the stomach, and the gastric stimulation hardware was confirmed.

**Fig. 1:**
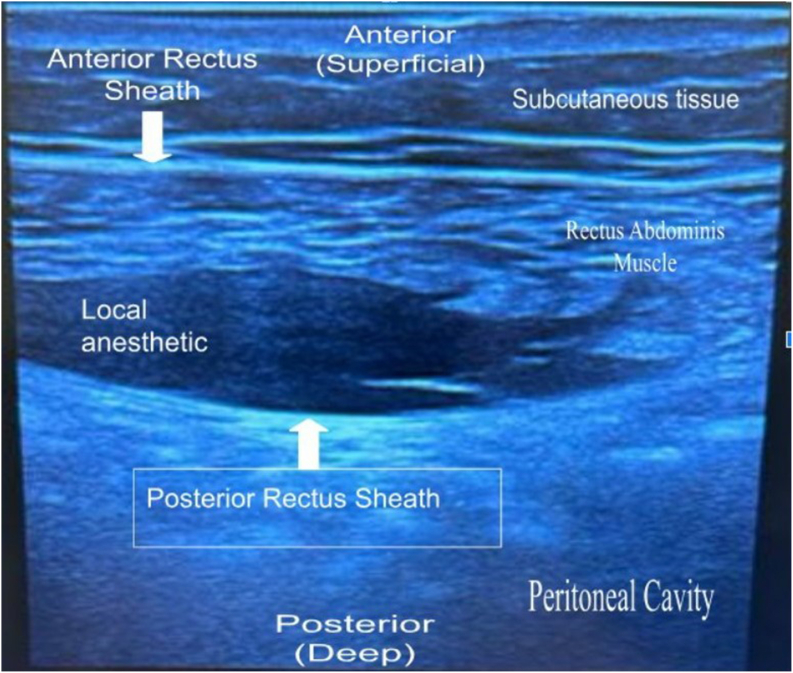
Diagnostic ultrasound-guided left upper rectus sheath nerve block depositing a mixture of 1 % lidocaine and 40 mg/mL of triamcinolone acetonide posterior to the left rectus abdominis muscle and superficial to the posterior rectus sheath to create a hydrodissection spread.

Given the clinical suspicion for ACNES, the patient underwent ultrasound-guided unilateral left rectus sheath block with 9 mL of 1 % lidocaine and 1 mL of 40 mg/mL of triamcinolone acetonide, which resulted in 80 % pain relief for two months. A subsequent nerve block with lidocaine and triamcinolone acetonide provided approximately similar pain relief for almost two months. (Fig. 1).

### 3.5 Treatment plan & outcome

**Fig. 2:**
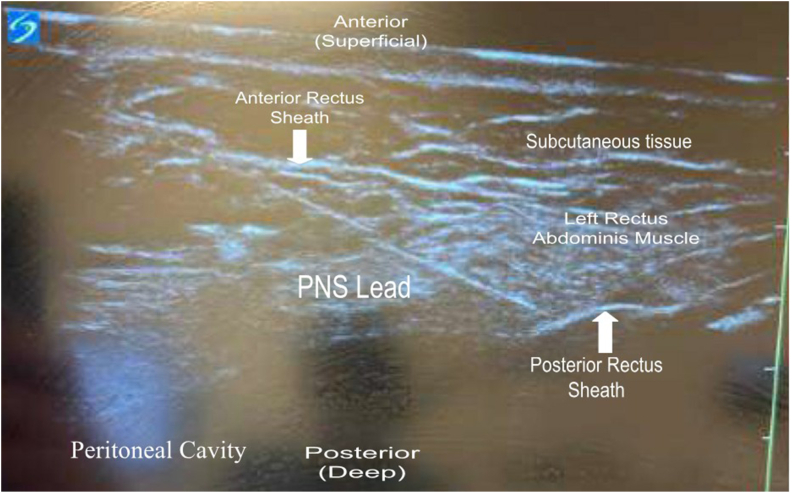
Peripheral nerve stimulator lead placement superficial to the posterior rectus sheath from a lateral to medial approach.

Clinical suspicion for ACNES was further increased based on the patient's response to the diagnostic nerve blocks, followed by the subsequent return of debilitating pain after the nerve blocks' effects wore off. We discussed the risks and benefits of corticosteroids, especially near superficial anatomical structures of the body, which could lead to soft tissue atrophy, fat necrosis, muscle tears/weakness along with tolerance to the effects of the corticosteroids. The collective decision was made to elect for placement of a temporary peripheral nerve stimulator targeting the left anterior cutaneous nerve for ACNES. The procedure was performed under a high-frequency linear ultrasound probe to identify the posterior rectus sheath, composed of the transverse abdominis aponeurosis and internal oblique underneath the rectus abdominis muscle. The peritoneal membrane and underlying bowel loops were also identified to ensure a safe needle trajectory. The skin around the planned entry point and the subcutaneous tissues were injected with 1 % lidocaine via a 25-G, 1.5-inch needle. Next, using ultrasound guidance, a percutaneous sleeve and stimulating probe lead introduction system was advanced from a lateral to medial direction employing an in-plane approach. The introducer needle was carefully advanced until it pierced the posterior aspect of the rectus abdominis muscle and was then positioned in the anatomic plane where the anterior cutaneous nerves are located: between the rectus abdominis muscle and the posterior rectus sheath. Electrical stimulation was used to elicit paresthesia in the patient's area of pain, confirming nerve target acquisition. The lead location was adjusted until optimal paresthesia overlap of the pain was achieved. The stimulating probe was removed from the introducer needle, and a percutaneous lead was guided through the needle and delivered to a location in similar proximity to the targeted nerve (Fig. 2).

## 4 Results

One week post-surgery, the patient reported an 80 % reduction in pain, decreasing from a 9/10 to a 2/10 on the numerical rating scale. This improvement was accompanied by a higher quality of life, including better sleep, the ability to play with her children, and walking for extended periods without assistance. Two months (60 days) per accordance with the device's FDA approval, the temporary peripheral nerve stimulator was removed. Her abdominal pain remained at a 2/10, and her quality of life continued to improve with the same outcomes at the six-month evaluation. At the eight-month mark, the patient developed a recurrence of symptoms and a discussion of the possibility of a permanent PNS implant.

## 5 Discussion

Chronic abdominal pain is a common and prevalent symptom that can arise from numerous underlying causes, making its diagnosis and treatment particularly challenging. Considering the critical need to exclude infectious, genitourinary, and vascular origins of abdominal pain that carry significant mortality risks, many clinicians understandably exhibit a bias towards ‘visceral thinking’ when evaluating patients with abdominal discomfort [3]. When life-threatening conditions have been ruled out, but continue to suffer from pain. Patients often continue an extensive diagnostic assessment, and are subject to delay in care, misallocation of healthcare resources, and even invasive surgical interventions, none of which reveal an underlying source of their pain. Some patients may receive erroneous diagnoses of functional gastrointestinal disorders, such as irritable bowel syndrome, while others might be diagnosed with psychiatric conditions like conversion disorder [17]. Interestingly, patients suffering from ACNES frequently experience concomitant vague visceral symptoms like nausea, bloating, and dyspepsia. A large retrospective study identifying characteristics among diagnosed patients with ACNES discovered 47 % of patients reported pseudovisceral complaints [19]. The author in this large retrospective study proposes the mechanism for these findings stem from the convergence of somatic and visceral C-fibers at the lamina I neurons in the spinal cord, and nerve irritation is interpreted by the brain as pseudovisceral symptoms [19].

Following a negative laboratory and imaging work-up, ACNES should always be considered in the differential diagnosis for patients experiencing chronic abdominal wall pain near the periumbilical region, specifically along the rectus abdominis muscle. If one has a high index of clinical suspicion and a positive rectus sheath block, one should pursue one of the most well-established and cost-effective treatment options involving trigger point injections with local anesthetic under ultrasound guidance at the site of maximal tenderness [2,4,7]. When it comes to interventional pain techniques and medication regimens, some physicians will utilize corticosteroids to prolong the duration of analgesia for a particular disease. However, Mol et al. found no statistical difference in adding corticosteroids (methylprednisolone) to lidocaine in the setting of ACNES for therapeutic relief at 6 and 12 weeks [21]. The authors conclude against the use of additive corticosteroids with local anesthetics, given the risk of soft tissue atrophy and systemic adverse effects, as many of these individuals receive numerous repeat injections given the limited treatment options [19]. In our case report, peripheral nerve stimulation (PNS) was chosen due to the unsustainability of continuing serial rectus sheath nerve block injections with local anesthetic and corticosteroid for achieving outcomes of 1–2 months of pain relief. The decision to proceed with PNS was made after discussing the patient's goals, which included achieving a more stable and prolonged period of analgesia. The cycle of recurring debilitating pain followed by short-term intervention was negatively impacting her quality of life. PNS offered the potential for a longer-lasting solution, avoiding the logistical, emotional, overall health burden of repeated corticosteroid injections.

Repeated exposure to corticosteroids in a young female patient could lead to potentially devastating implications in regards to bone, cardiovascular, skin, hormonal, and metabolic health. There have been no study comparisons among the most effective ultrasound guided techniques pertaining to trigger point injections around the site of entrapment, rectus sheath block, or transverse abdominis plane block for therapeutic purposes, but regardless of the technique, their analgesic effects are not sustained beyond 1–2 months [2,10].

Today, PNS is an emerging neuromodulation technique that offers promising alternatives for managing chronic and neuropathic pain, particularly in patients unresponsive to conventional therapies. PNS modulates pain signaling pathways by delivering electrical impulses to targeted peripheral nerves, providing relief in various conditions such as post-amputation pain, chronic low back pain, and peripheral neuropathies [13]. PNS alleviates chronic pain by modulating both peripheral and central nervous system pathways. Although the exact mechanism is not fully understood, authors have proposed that orthodromic stimulation of non nociceptive Aβ nerve fibers results in the activation of the interneurons of the superficial layers in the dorsal horn of the spinal cord, the same interneurons that are involved in the processing and transmission of nociceptive information by peripheral small-diameter Aδ and C fibers. This nonpainful stimulation provided by PNS inhibits these interneurons leading to interruption of pain signals [[13], [14], [15],^18^]. In 2017, Chrona at el described a comprehensive literature review involving the available treatment options for pain management of ACNES ranging from systemic drug administration, trigger point injections, ultrasound-guide blocks, chemical neurolysis, radiofrequency ablation botulinum toxin injections, and neurectomies [2]. At that moment in time, the paper describes how there was no current available literature or cases published on neuromodulation techniques for the therapy of ACNES, but it may have a role in the future for treating neuropathic conditions. We present the third potentially reported case and demonstrate the effectiveness of PNS for ACNES.

Data regarding the outcomes of PNS treatment for ACNES are limited to two case reports with two cases exhibiting limited information on the diagnostic criteria, technique utilized, and outcomes from these studies. No prospective studies were identified in this comprehensive search. In 2024, the Amorizzo at el study described the patient's outcome at two year follow up period as “healthy” [16]. The authors were not able to extrapolate any meaningful numerical data related to outcomes from their PNS treatment option. In 2022, Ferreira-Silva et al. published the first documented report of a 23-year-old female who suffered from bilateral anterior abdominal wall pain secondary to ACNES treated with two percutaneous permanent PNS leads on each side of the abdomen at a frequency of 100Hz [18]. The authors reported a decrease from 9 to 4 on the numeric rating scale leading to 55 % pain reduction score at the 6 month follow up. In comparison to our patient from the previous two studies, the patient reported a 78 % pain reduction from a baseline visual analog scale (VAS) of 9 out of 10 to 2 out of 10 with returning symptoms at the 8 month evaluation after placement of a temporary PNS device for 60 days.

Further illustrating the emerging interest in PNS for abdominal wall pain, other researchers have explored different anatomical targets for chronic abdominal wall pain. Instead of isolating the anterior cutaneous nerve at its specific entrapment point, some recent studies focus on stimulating the thoracoabdominal nerves more proximally within the transversus abdominis plane (TAP). For example, Lam et al. detailed a novel medial-to-lateral ultrasound-guided technique for placing PNS leads via a TAP approach to manage abdominal wall pain. The authors hypothesized that positioning the leads between the internal oblique and transverse abdominis muscles would reduce lead migration compared to placing them directly through the muscle belly. At the same time, placing the lead with multiple contracts at a more proximal region of the intercostal nerve will allow for potential greater coverage of the targeted nerves and surrounding nerves such as the iliohypogastric or ilioinguinal hypothesized by the authors of this study [24]. Similarly, a 2024 case series by Eden et al. reported on the use of multicontact PNS systems placed within the TAP for patients with chronic abdominal wall pain refractory to conservative management [22]. These methods aim for broader neuromodulation of the T7-T12 intercostal nerves as they travel through the fascial plane, which differs from a strategy of targeting the distal anterior cutaneous branch specifically at the border of the rectus muscle. The one retrospective case series by Eden et al. demonstrated 3 out of the 4 participants had greater than 50>% for over one year with permanent PNS implants following a positive TAP block and positive PNS trial through this approach.

In conclusion, while this case report adds to the nascent body of evidence suggesting peripheral nerve stimulation may be a viable option for ACNES, its limitations must be acknowledged. The limitations of these studies are primarily due to their small scale and the absence of control groups. These factors hinder the ability to draw definitive conclusions about the efficacy of the interventions being studied. Therefore, further research is needed and suggesting future prospective studies could incorporate control groups, such as a sham PNS procedure or a group randomized to receive serial rectus sheath blocks, to more rigorously evaluate the efficacy of PNS against other treatment modalities. Such studies with standardized outcomes are needed to establish more definitive outcomes and standardized techniques for PNS treatment in ACNES.

## 6 Conclusion

Overall, this case report describes the successful application of PNS for anterior cutaneous nerve entrapment syndrome in a 38-year-old female with a complex history of multiple gastrointestinal surgeries. While this study and prior case reports show encouraging outcomes of PNS treatment for ACNES, more extensive research is needed to confirm its long-term effectiveness and optimal application due to a lack of high quality data. This case contributes to the growing evidence supporting the potential of PNS in treating this challenging chronic abdominal condition.

## Informed consent

Consent was obtained from the participant in this case report.

## Financial disclosures

None of the authors involved in the creation of this case report have identified any competing interests and have no sources of funding to declare for this manuscript.

## Declaration of competing interest

The authors declare that they have no known competing financial interests or personal relationships that could have appeared to influence the work reported in this paper.
